# The Release Kinetics of Eosinophil Peroxidase and Mitochondrial DNA Is Different in Association with Eosinophil Extracellular Trap Formation

**DOI:** 10.3390/cells10020306

**Published:** 2021-02-03

**Authors:** Nina Germic, Timothée Fettrelet, Darko Stojkov, Aref Hosseini, Michael P. Horn, Alexander Karaulov, Dagmar Simon, Shida Yousefi, Hans-Uwe Simon

**Affiliations:** 1 Institute of Pharmacology, University of Bern, Inselspital, INO-F, CH-3010 Bern, Switzerland; nina.germic@pki.unibe.ch (N.G.); timothee.fettrelet@unil.ch (T.F.); darko.stojkov@pki.unibe.ch (D.S.); aref.hosseini@pki.unibe.ch (A.H.); shida.yousefi@pki.unibe.ch (S.Y.); 2 Department of Biochemistry, University of Lausanne, CH-1066 Epalinges, Switzerland; 3 Department of Clinical Chemistry, Inselspital, Bern University Hospital, University of Bern, CH-3010 Bern, Switzerland; Michael.Horn@insel.ch; 4 Department of Clinical Immunology and Allergology, Sechenov University, 119991 Moscow, Russia; drkaraulov@mail.ru; 5 Department of Dermatology, Inselspital, Bern University Hospital, University of Bern, CH-3010 Bern, Switzerland; dagmar.simon@insel.ch; 6 Laboratory of Molecular Immunology, Institute of Fundamental Medicine and Biology, Kazan Federal University, 420012 Kazan, Russia

**Keywords:** degranulation, eosinophils, eosinophil extracellular traps, eosinophil peroxidase, kinetics, mitochondrial DNA

## Abstract

Eosinophils are a subset of granulocytes characterized by a high abundance of specific granules in their cytoplasm. To act as effector cells, eosinophils degranulate and form eosinophil extracellular traps (EETs), which contain double-stranded DNA (dsDNA) co-localized with granule proteins. The exact molecular mechanism of EET formation remains unknown. Although the term “EET release” has been used in scientific reports, it is unclear whether EETs are pre-formed in eosinophils and subsequently released. Moreover, although eosinophil degranulation has been extensively studied, a precise time-course of granule protein release has not been reported until now. In this study, we investigated the time-dependent release of eosinophil peroxidase (EPX) and mitochondrial DNA (mtDNA) following activation of both human and mouse eosinophils. Unexpectedly, maximal degranulation was already observed within 1 min with no further change upon complement factor 5 (C5a) stimulation of interleukin-5 (IL-5) or granulocyte/macrophage colony-stimulating factor (GM-CSF)-primed eosinophils. In contrast, bulk mtDNA release in the same eosinophil populations occurred much slower and reached maximal levels between 30 and 60 min. Although no single-cell analyses have been performed, these data suggest that the molecular pathways leading to degranulation and mtDNA release are at least partially different. Moreover, based on these data, it is likely that the association between the mtDNA scaffold and granule proteins in the process of EET formation occurs in the extracellular space.

## 1. Introduction

Eosinophils are a subpopulation of granulocytes that was identified in 1879 by Paul Ehrlich based on the capacity of their granules to be stained with acidic dye eosin [1]. Eosinophil-specific granules are membrane-bound organelles consisting of a crystalloid core surrounded by a matrix and represent the fundamental morphological characteristic of mature eosinophils [2]. Large amounts of four cationic proteins are preformed and stored within specific granules: major basic protein (MBP), eosinophil peroxidase (EPX), eosinophil cationic protein (ECP) and eosinophil-derived neurotoxin (EDN) [3,4]. Eosinophils are powerful secretory cells, and the release of granule proteins is essential for their function as immune effector cells [1]. Different pathways of eosinophil degranulation have been described. Piecemeal degranulation (PMD) is the most prevalent physiological pathway of eosinophil degranulation [5,6]. During PMD, eosinophil-specific granules undergo progressive depletion of their content, which is transferred to the cell surface by eosinophil Sombrero vesicles (EoSVs), enabling the differential release of eosinophil mediators [7,8]. Exocytosis occurs following granule–granule fusion and subsequent merging with the plasma membrane, resulting in the formation of open channels for granule content release [9,10]. Eosinophils can undergo cytolysis, characterized by disintegration of nuclear and plasma membranes together with the release of intact granules, which accumulate in extracellular space as clusters of free eosinophil granules [11]. As seen during exocytosis, granule fusion was also observed in the process of eosinophil cytolysis [12].

Viable eosinophils are also able to exert effector functions in host defense by forming eosinophil extracellular traps (EETs), which consist in mitochondrial DNA (mtDNA) and granule proteins released from activated cells [13,14]. Localized activities of toxic granule proteins associated with mtDNA scaffold result in an efficient antimicrobial defense and limited damage to surrounding host tissues [15]. As part of the innate immune response, EETs are capable of capturing and killing bacteria [16,17,18,19]. Similar extracellular trap formation can be formed by other cells, e.g., neutrophils [20], basophils [21], mast cells [22], monocytes [23] and tissue macrophages [24,25]. In contrast to neutrophil extracellular trap (NET) formation [15], little is known about the mechanism of EET formation. In this context, it should be mentioned that a lytic mechanism underlining the release of EETs from nuclear origin has been reported [26], although the issue whether cell death is required or not for EET and NET formation is a matter of scientific dispute [15,27].

In this study, we aimed to define the kinetics of degranulation and concomitant mtDNA release by both human and mouse eosinophils in vitro. Unexpectedly, degranulation occurred much more rapidly compared to mtDNA release, suggesting that these two functional responses are initially independent and rely on different molecular pathways. Therefore, EETs seem to be formed over time in the extracellular space. While granule proteins are rapidly available, the release of the mtDNA is limited immediately after eosinophil activation.

## 2. Materials and Methods

### 2.1. Reagents

EasySep Human Eosinophil Isolation Kit and EasySep Mouse PE Positive Selection Kit were obtained from StemCell Technologies (Cologne, Germany). Human interleukin-5 (IL-5), human interferon-γ (IFN-γ), human eotaxin/CCL11 and mouse granulocyte/macrophage colony-stimulating factor (GM-CSF) were purchased from R & D Systems (Abingdon, UK). Human GM-CSF was purchased from Novartis Pharma (Nuremberg, Germany). Human and mouse complement factor 5a (C5a) were supplied from Hycult Biotech (Uden, The Netherlands). Pancoll Human was from PAN-Biotech (Aidenbach, Germany). Immobilon Forte Western HRP substrate, phorbol 12-myristate 13-acetate (PMA), platelet-activating factor (PAF) and Hemacolor Rapid staining kit were purchased from Merck Millipore (Darmstadt, Germany). Ionomycin was supplied from LKT Laboratories (St Paul, MN, USA). Dulbecco’s Phosphate Buffered Saline (PBS), RPMI-1640 medium, Histopaque 1119, bovine serum albumin (BSA), cytochalasin B, saponin, Triton X-100, protease inhibitor cocktail, o-phenylenediamine (OPD) dihydrochloride and hydrogen peroxide (H_2_O_2_) solution were purchased from Sigma-Aldrich (Buchs, Switzerland). MitoTracker Deep Red FM, MitoSOX Red, SYTOX Orange, Prolong Gold Antifade mounting medium, Hoechst 33342, EDTA (pH 8.0), Pierce BCA protein assay kit and Quant-iT PicoGreen dsDNA assay kit were obtained from ThermoFisher Scientific (distributed by LuBioScience, Lucerne, Switzerland). Medium 199 and X-VIVO 15 medium were purchased from Lonza (Walkersville, MD, USA). Proteinase K was purchased from Roche Diagnostics (Rotkreuz, Switzerland). Deoxyribonuclease I (DNase I) was from Worthington Biochemical Corporation (Lakewood, NJ, USA). Polyvalent human IgG was a kind gift from CSL Behring (Bern, Switzerland). Normal goat sera were obtained from Vector Laboratories (Burlingame, CA, USA). Normal donkey sera were from Milan Analytica AG (Rheinfelden, Switzerland). Black, glass-bottom 96-well plates were from Greiner Bio-One (Frickenhausen, Germany). Fetal calf serum (FCS), HRP-conjugated secondary antibodies and Hyperfilm ECL were from GE Healthcare Life Sciences (Little Chalfont, UK).

### 2.2. Purification of Human Eosinophils

Human blood eosinophils from healthy individuals were purified as described previously [17]. Briefly, white blood cells were separated by density-gradient centrifugation at 800× *g* for 20 min using Pancoll Human (density of 1.077 g/mL) (PAN-Biotech). The upper phase containing peripheral blood mononuclear cells (PBMCs) was removed, while the erythrocytes in the lower phase were treated with lysis solution (155 mM NH_4_Cl, 10 mM KHCO_3_ and 0.1 mM EDTA) to obtain granulocytes. Isolation of eosinophils was performed by a negative selection procedure using an EasySep Human Eosinophil Isolation Kit (StemCell Technologies). Purity of eosinophils was ≥97% as assessed by Hemacolor Rapid staining kit (Merck Millipore) followed by light microscopic analysis.

Written, informed consent was obtained from all blood donors. The Ethics Committee of the Canton of Bern approved this study.

### 2.3. Purification of Mouse Eosinophils

*Il5* (IL-5) transgenic mice overexpressing IL-5 in CD3^+^ T cells (Tg(Cd3d-Il5)NJ.1638Nal) [28] were kindly provided by J. J. Lee (Mayo Clinic, Scottsdale, AZ, USA). Mouse eosinophils were isolated from the bone marrow and peripheral blood of *Il5*^tg^ mice between 8 and 16 weeks of age. As reported earlier [29], bone marrow cells were collected by flushing the femurs, tibia, and humerus with isolation medium (2% FCS in PBS), using a 26-gauge needle, and filtering through a sterile 70 µm nylon cell strainer. Bone marrow single-cell suspensions were washed with isolation medium and resuspended at a concentration of 1 × 10^8^ cells/mL. T cells, B cells, macrophages and neutrophils were depleted with PE-labelled antibodies against CD8α, CD19, CD90.2, and Ly-6G (Miltenyi Biotec, Bergisch Gladbach, Germany), respectively, using an EasySep Mouse PE Positive Selection Kit (StemCell Technologies). Mouse eosinophil purity was 90ߝ95% as assessed by staining with the Hemacolor Rapid staining kit (Merck Millipore) followed by light microscopic analysis.

Eosinophils from the peripheral blood were isolated as previously described [30]. In brief, peripheral blood was layered on top of Histopaque 1119 (density of 1.119 g/mL) (Sigma-Aldrich) and centrifuged at 800× *g* for 20 min. The eosinophil-containing layer was treated for 30 sec with ice-cold distilled water to lyse any remaining red blood cells and washed with PBS. Cells were resuspended at a concentration of 1 × 10^8^ cells/mL in isolation medium and eosinophils were isolated using an EasySep Mouse PE Positive Selection Kit (StemCell Technologies) as described above.

The study was approved by the Veterinary Office of the Canton of Bern and conducted in accordance with Swiss federal legislation on animal welfare (BE 49/18).

### 2.4. Activation of Eosinophils

Isolated human eosinophils were primed with 25 ng/mL IL-5, 25 ng/mL IFN-γ or 100 ng/mL GM-CSF for 20 min. Cells were subsequently stimulated with 10 nM C5a, 100 nM PAF or 10 ng/mL eotaxin for the indicated times. Unprimed eosinophils were also treated with 25 nM PMA or 1 µM ionomycin for 15 min.

Plasma membrane permeability was tested using SYTOX Orange uptake assay. Eosinophils (1 × 10^5^/50 µL X-VIVO 15 medium) were placed in triplicates in black, glass-bottom 96-well plates (Greiner Bio-One) in the presence and absence of IL-5 (25 ng/mL) for 20 min. Medium with or without C5a (10 nM) was then added in the presence of 0.5 µM SYTOX Orange. Triton X-100 (1%) was used as a control to permeabilize the plasma membrane. The plate was placed in a spectrofluorometer (SpectraMax M2, Molecular Devices, Biberach an der Riss, Germany) equipped with 37 °C incubator. The fluorescent dye was excited at 547 nm and the fluorescence emission intensity was measured at 570 nm every 10 min with the bottom-read option until the end of the experiment.

Mouse eosinophils were purified from *Il5*^tg^ mice and cultured in X-VIVO 15 medium for 1 h at 37 °C to decrease the activation state of eosinophils following sustained IL-5 priming in *Il5*^tg^ mice under in vivo conditions. Eosinophils were then washed and primed with 100 ng/mL GM-CSF for 20 min followed by activation with 100 nM C5a in a time-dependent manner.

### 2.5. Degranulation Assay

Colorimetric detection of eosinophil peroxidase (EPX) in the supernatants of stimulated cells was determined as reported previously [31]. Freshly purified eosinophils were resuspended (0.15 × 10^6^/150 µL X-VIVO 15 medium) and stimulated as described above. Cytochalasin B (5 µM) was added to the cell suspension in the final 5 min of priming. Unprimed eosinophils were also treated with cytochalasin B, which was added 5 min prior to stimulation with 25 nM PMA or 1 µM ionomycin. At the end of the incubation time, 150 µL of o-phenylenediamine (OPD) substrate was added to the cells. Substrate solution was prepared by adding 800 µL of 5 mM OPD to 4 mL 1 M Tris (pH 8.0), 1.25 µL 30% H_2_O_2_ and 5.2 mL distilled water. To determine the total amount of EPX, eosinophils were lysed with 0.2% Triton X-100. Cell suspension was transferred into a black, glass-bottom 96-well plate and the absorbance was measured at 492 nm (SpectraMax M2, Molecular Devices).

Degranulation analysis was also performed by flow cytometry. The plasma membrane expression of surrogate marker CD63 [32] was measured using the APC-conjugated mouse anti-human CD63 antibody (clone H5C6, #353008; BioLegend, London, UK) or PE-conjugated rat anti-mouse CD63 antibody (clone NVG-2, #564222; BD Biosciences, San Jose, CA, USA). Cells were fixed in BD CellFix solution (BD Biosciences), acquired by flow cytometer (FACSVerse, BD Biosciences) and analyzed using FlowJo software (Tree Star Inc., Ashland, OR, USA).

### 2.6. Confocal Laser Scanning Microscopy

Freshly purified human (0.25 × 10^6^/100 µL medium 199) and mouse eosinophils (0.25 × 10^6^/100 µL X-VIVO 15 medium) were seeded on 12 mm glass coverslips, primed for 20 min with IL-5 or GM-CSF, and stimulated with C5a for the indicated times. Staining with cell-permeable fluorescent dyes Mitotracker Deep Red (33 nM) or MitoSOX Red (5 µM) was performed in live cells before fixation, according to the corresponding manufacturer’s instructions. Cells were fixed with 4% paraformaldehyde for 5 min, washed with PBS (pH 7.4), and permeabilized with 0.05% saponin in PBS (pH 7.4) for 5 min at room temperature (RT). Subsequently, the immunofluorescence staining was performed in the presence of 0.01% saponin. Nonspecific binding was prevented by incubation of cells in blocking buffer (containing human immunoglobulins, secondary antibody species serum, and 7.5% BSA) at RT for 10 min. Primary monoclonal mouse anti-mouse EPX antibody (1:400, clone MM25-82.2; obtained from Lee Laboratories, Mayo Clinic, Scottsdale, AZ, USA), monoclonal rabbit anti-human CD45 antibody (1:100, clone EP322Y, #ab40763; Abcam, Cambridge, UK) and monoclonal rat anti-mouse Siglec-F antibody (1:600, clone E50-2440, #552125; BD Biosciences) were diluted in blocking solution and incubated with the cells at RT for 2 h. Thereafter, the following secondary antibodies, namely goat anti-mouse Alexa Fluor 488 (1:400; ThermoFisher Scientific), goat anti-rabbit Alexa Fluor 532 (1:400; ThermoFisher Scientific) or donkey anti-rat PE (1:200; Jackson ImmunoResearch, Ely, UK) were incubated at RT for 1 h. For controls, cells were stained with secondary antibody only. Cells were subsequently washed in PBS (pH 7.4), stained with Hoechst 33,342 (1 µg/mL) for an additional 10 min and mounted with Prolong Gold Antifade mounting medium.

Slides were examined and image acquisition was performed by confocal laser scanning microscope LSM 800 (Carl Zeiss Micro Imaging, Jena, Germany) using a Plan-Apochromat 63×/1.40 Oil DIC objective. The mean fluorescence intensity (MFI) of EPX (green channel) was quantified within the cells, which were delimited using “Spots” or “Surfaces“ mode in Imaris software (Bitplane AG, Zurich, Switzerland). For better visualization of EETs, the gamma correction function together with min/max thresholds of Imaris software was used to optimize the image display by intensifying the grey value of red (mtDNA) and green (EPX) channels.

### 2.7. Quantification of Released dsDNA in Culture Supernatants

Released dsDNA in culture supernatants was quantified as previously reported [33]. In brief, eosinophils (1 × 10^6^/500 µL X-VIVO 15 medium) were stimulated as described above. In the final 2 min of the incubation time, low concentrations of 0.2 mg/mL proteinase K (Roche Diagnostics) and 2.5 U/mL DNase I (Worthington Biochemical Corporation) were added. Reactions were stopped by the addition of 2.5 mM EDTA [pH 8.0]. Cells were centrifuged at 13,000 rpm for 5 min. One hundred microliters of supernatant was transferred to black, glass-bottom 96-well plates (Greiner Bio-One). The fluorescent activity of PicoGreen dye bound to dsDNA was excited at 502 nm, and the fluorescence emission intensity was measured at 523 nm by using a spectrofluorometer (SpectraMax M2, Molecular Devices), according to the instructions described in the Quant-iT PicoGreen dsDNA assay kit (ThermoFisher Scientific). Mouse eosinophils were treated with 0.2 mg/mL proteinase K and 2.5 U/mL DNase I for 5 min prior to 1 h culture in X-VIVO 15 medium to remove any dsDNA released from mouse eosinophils owing to sustained IL-5 priming in *Il5*^tg^ mice under in vivo conditions.

### 2.8. Immunoblotting

Protein expression of intracellular and released EPX was analyzed by immunoblotting. Human eosinophils (2 × 10^6^ in RPMI-1640 supplemented with 5% FCS) were stimulated as described above. At the end of the incubation time, eosinophils were put on ice and centrifuged at 1800 rpm for 7 min at 4 °C.

After centrifugation, the cell pellet was washed with ice-cold PBS and proteins were lysed in lysis buffer (50 mM Tris (pH 7.4), 150 mM NaCl, 10% glycerol, 1% Triton X-100, 2 mM EDTA, 10 mM NaPyrophosphate, 50 mM NaF and 200 μM Na_3_VO_4_). Shortly before use, a protease inhibitor cocktail (Sigma-Aldrich) and 1 mM PMSF were added to the lysis buffer. Cells were incubated for 25 min on ice, and protein lysates were collected after high-speed centrifugation (13,300 rpm for 15 min at 4 °C).

For detection of released EPX in the supernatants of stimulated eosinophils, four volumes of ice-cold acetone (containing 20 mM DTT) were added to one volume of the collected supernatant. The mixture was vortexed thoroughly and incubated at −80 °C overnight. On the next day, tubes were centrifuged at 10,000× *g* for 15 min at 4 °C. The cell pellet was air-dried and resuspended in lysis buffer as described above.

Protein concentrations were determined by BCA protein assay kit (ThermoFisher Scientific) and the lysates were heated at 95 °C for 5 min. Fifty micrograms of denatured proteins were loaded on 12% SERVA*Gel* TG PRiME gel (SERVA Electrophoresis, Heidelberg, Germany), followed by protein transfer on Immobilon-P PVDF transfer membrane (Merck Millipore). The membranes were blocked in 5% non-fat dry milk in TBST (0.1% Tween-20 in 20 mM Tris and 150 mM NaCl (pH 7.6)) followed by incubation with the monoclonal mouse anti-EPX antibody (1:1000, clone MM25-82.2; obtained from Lee Laboratories) or monoclonal mouse anti-GAPDH antibody (1:5000, clone 6C5, #MAB374; Merck Millipore). After the addition of HRP-coupled secondary antibodies (1:5000; GE Healthcare Life Sciences), the signal was detected by chemiluminescence using the Immobilon Forte Western HRP substrate (Merck Millipore) on photosensitive Hyperfilm ECL (GE Healthcare Life Sciences).

### 2.9. Statistical Analysis

Data were analyzed using GraphPad Prism 8 software (GraphPad Software Inc., La Jolla, CA, USA) and presented as mean values ± SEM. To compare groups, one-way (quantification of dsDNA release and degranulation assays) and two-way ANOVA (viability assay) were applied. The mean of each condition was compared to the mean of the “Medium” condition. *p* values < 0.05 were considered statistically significant.

## 3. Results

### 3.1. High Efficacy of C5a in Stimulating Degranulation in Cytokine-Primed Human Eosinophils

**Figure 1:**
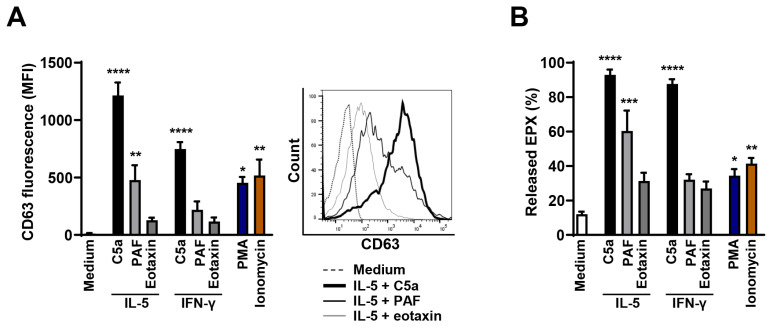
Degranulation of human eosinophils following different stimuli. (**A**) Flow cytometry. Human eosinophils were primed with IL-5 or IFN-γ for 20 min, and subsequently stimulated with C5a, PAF, or eotaxin for 15 min. Unprimed eosinophils were incubated with PMA or ionomycin for 15 min. Eosinophil degranulation was assessed by CD63 surface expression by flow cytometry (*n* = 3). Right: A representative histogram of flow cytometry data is shown. (**B**) EPX assay. Human eosinophils were treated as described in (A). Released eosinophil granule protein EPX in the cell supernatant was measured by absorbance at 492 nm (*n* = 3). Values are means ± SEM. * *p* < 0.05; ** *p* < 0.01; *** *p* < 0.001; **** *p* < 0.0001.

We investigated the effects of different physiological (C5a, PAF, eotaxin) stimuli on the cytokine-primed [34] and non-physiological (PMA [35], ionomycin [30]) agonists on unprimed eosinophils. IL-5 priming has been reported to increase eosinophil granule protein release [36]. The extent of eosinophil degranulation was measured by the cell surface expression of CD63, which is predominantly located on the membranes of eosinophil-specific granules [32]. A significant increase in the surface expression of CD63 was observed after C5a and, to a lesser extent, also after PAF stimulation following IL-5 priming. In contrast, eotaxin did not induce a significant CD63 upregulation independent of priming factors. Among non-physiological stimuli, PMA and ionomycin also induced a significant increase in CD63 surface expression, although the efficacy of these compounds was reduced compared to C5a (Figure 1A).

To further quantify the extent of eosinophil degranulation, we measured the released amounts of the granule protein EPX in eosinophil supernatants (Figure 1B). IL-5 or IFN-γ-primed and C5a-stimulated eosinophils demonstrated a significant release of EPX. PAF stimulation of IL-5-primed eosinophils also resulted in significant EPX release. In contrast, eotaxin had no effect in this system. These results support the assumption that C5a and PAF, but not eotaxin, can trigger degranulation of cytokine-primed eosinophils. Moreover, PMA and ionomycin also induced significant EPX release, but with reduced efficacy compared to C5a (Figure 1B), supporting the CD63 expression data (Figure 1A). In agreement with these findings, PMA has previously been reported to stimulate less efficiently granule protein release compared to C5a [37]. In addition, higher concentration and longer incubation time were required for mouse eosinophils to release MBP upon PMA stimulation [38]. Based on these data, we decided to use the combined IL-5/C5a treatment of human eosinophils for all subsequent functional in vitro assays.

### 3.2. Immediate EPX and Delayed mtDNA Release of IL-5-Primed and C5a-Stimulated Human Eosinophils

**Figure 2:**
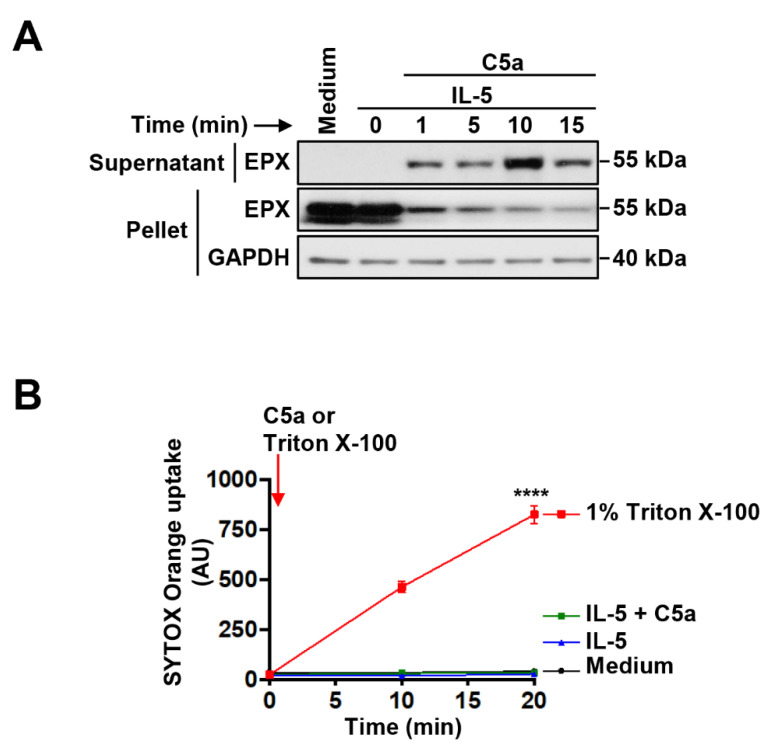
Time-dependent EPX release by viable human eosinophils. Human eosinophils were primed with IL-5 and subsequently stimulated with C5a for the indicated times. (**A**) The presence of EPX in the cell pellet and the supernatant fraction was detected by immunoblotting with monoclonal mouse anti-EPX antibody. GAPDH protein levels were analyzed as loading controls. A representative immunoblot of two independent experiments is shown. (**B**) Viability assay. Activated human eosinophils exhibited no SYTOX Orange uptake, indicating intact plasma membrane during the entire period of the experiment. **** *p* < 0.0001.

To determine the kinetics of C5a-induced eosinophil degranulation, we assessed the release of EPX in a time-dependent manner using immunoblotting (Figure 2A). Unexpectedly, we detected released EPX in the cell supernatant already 1 min after C5a stimulation. Simultaneously, we observed a strong decrease of the intracellular EPX content (Figure 2A). To confirm the viability of the eosinophils upon IL-5 priming and C5a activation, we measured the influx of SYTOX Orange. As a positive control, we used 1% Triton X-100 known to damage the plasma membrane. No plasma membrane permeabilization was detected in IL-5-primed and C5a-stimulated eosinophils during the entire experiment (Figure 2B), confirming previously published work [13].

**Figure 3:**
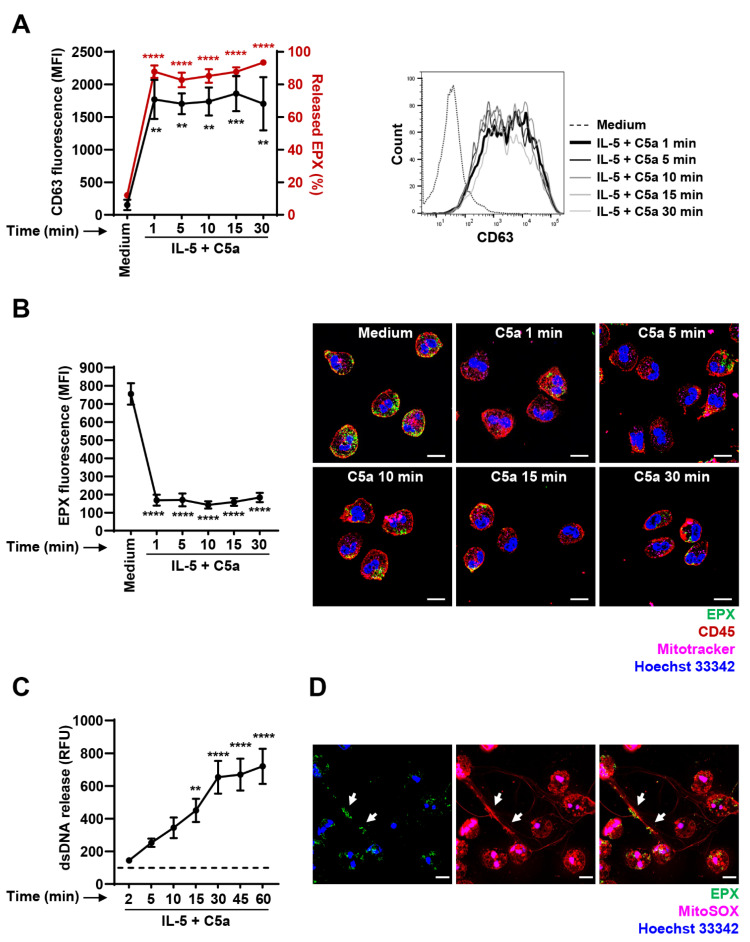
Time-dependent degranulation and extracellular trap (EET) formation by human eosinophils. (**A**) Degranulation assays. Isolated human eosinophils were primed with IL-5 and stimulated with C5a in a time-dependent manner (1–30 min). Eosinophil degranulation was assessed by CD63 surface expression using flow cytometry (black line) (*n* = 4) and quantification of released EPX (red line) (*n* = 3). Right: A representative histogram of flow cytometry data is shown. (**B**) Confocal microscopy. Freshly isolated human eosinophils were primed with IL-5 and stimulated with C5a for the indicated times (1–30 min). Mitochondria, eosinophil granule protein EPX, plasma membrane and nuclei were stained using Mitotracker Deep Red, monoclonal mouse anti-EPX antibody, monoclonal rabbit anti-CD45 antibody and Hoechst 33342, respectively. Left: Quantification of EPX mean fluorescence intensity (MFI). Cells were delimited using “Surfaces” mode in Imaris followed by the intracellular EPX (green channel) MFI quantification (*n* ≥ 11 cells per condition). Right: Representative images of eosinophil degranulation at the indicated time points are shown. Bars, 10 µm. (**C**) dsDNA release assay. Quantification of released dsDNA in supernatants of activated human eosinophils was assessed using PicoGreen fluorescent dye following the IL-5 priming and C5a activation in a time-dependent manner (*n* = 5). The dashed line indicates the mean level of spontaneously released dsDNA after 60 min eosinophil culture in the absence of stimulation. (**D**) Confocal microscopy. Human eosinophils were primed with IL-5 and activated with C5a for 15 min. A representative image of EET formation, indicated by white arrows, is shown. Mitochondria, EPX and nuclei were stained with MitoSOX Red, monoclonal mouse anti-EPX antibody and Hoechst 33342, respectively. For better visualization of EETs, the gamma correction function together with min/max thresholds of Imaris software was used to optimize the image display by intensifying the grey value of red (mtDNA) and green (EPX) channels. Bars, 10 µm. Values are means ± SEM. ** *p* < 0.01; *** *p* < 0.001; **** *p* < 0.0001.

To confirm the rapid degranulation after C5a stimulation of cytokine-primed eosinophils, we investigated the kinetics of eosinophil degranulation in more detail using CD63 surface expression and quantitative EPX release analyses. We observed strong surface CD63 upregulation and EPX release in the cell supernatant already after 1 min of C5a stimulation (Figure 3A). No evidence for additional degranulation was obtained within a time period of 30 min (Figure 3A). We also quantified the amount of intracellular EPX by confocal microscopy. Eosinophils were isolated from human blood, primed with IL-5 and activated with C5a in a time-dependent manner. Mitochondria, EPX, cell membrane and the nucleus were stained with Mitotracker, anti-EPX antibody, anti-CD45 antibody and Hoechst 33342, respectively. To assess the amount of intracellular EPX, we quantified its mean fluorescence intensity (MFI) using Imaris software. We observed again a maximal release of EPX within 1 min of C5a stimulation, and no evidence for additional EPX release was obtained at later time points (Figure 3B). Collectively, we demonstrated that C5a-induced degranulation of human eosinophils occurs within 1 min. Less than 20% of the intracellular EPX levels remained in the granules following eosinophil stimulation.

EETs contain an mtDNA scaffold, and it has been demonstrated that single eosinophils release mtDNA in a catapult-like manner [13]. To compare the kinetics of degranulation and mtDNA release, we performed the same time-dependent stimulation experiments and quantified mtDNA release from human eosinophils. Although some eosinophils release DNA rapidly, a significant mtDNA release of the entire eosinophil population was not reached before 15 min after C5a stimulation (Figure 3C), supporting previously reported data [13]. In contrast to the eosinophil degranulation pattern, a further increase in mtDNA release occurred up to 30–60 min (Figure 3C).

To demonstrate the presence of EETs following IL-5 priming and subsequent C5a stimulation, we stained eosinophils with anti-EPX antibody and MitoSOX. A representative confocal microscopy image of human EET (Figure 3D) shows co-localization (white arrows) of released EPX (green) and mtDNA (red), demonstrating extracellular co-localization of EPX and the mtDNA scaffold. Therefore, although we observed different kinetics of EPX and mtDNA release, an association occurs in the extracellular space, most likely owing to the physicochemical properties of both partners [20].

### 3.3. Immediate EPX and Delayed mtDNA Release of GM-CSF—Primed and C5a-Stimulated Mouse Eosinophils

**Figure 4:**
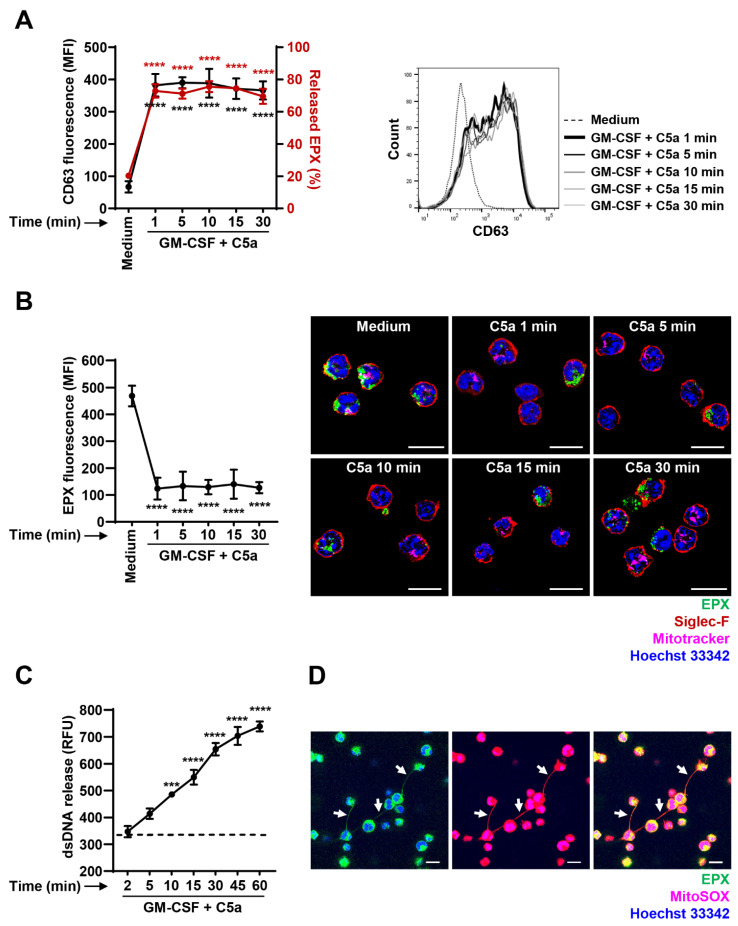
Time-dependent degranulation and EET formation by mouse eosinophils. (**A**) Degranulation assays. Isolated mouse eosinophils were primed with GM-CSF and stimulated in a time-dependent manner (1–30 min). Eosinophil degranulation was assessed by CD63 surface expression using flow cytometry (black line) (*n* = 3) and quantification of released EPX (red line) (*n* = 3). Right: A representative histogram of flow cytometry data is shown. (**B**) Confocal microscopy. Freshly purified mouse eosinophils were primed with GM-CSF and stimulated with C5a in a time-dependent manner (1–30 min). Mitochondria, eosinophil granule protein EPX, plasma membrane and nuclei were stained using Mitotracker Deep Red, monoclonal mouse anti-EPX antibody, monoclonal rat anti-Siglec-F antibody and Hoechst 33342, respectively. Left: Quantification of EPX mean fluorescence intensity (MFI). Cells were delimited using “Spots” mode in Imaris followed by the intracellular EPX (green channel) MFI quantification (*n* ≥ 11 cells per condition). Right: Representative images of eosinophil degranulation at the indicated time points are shown. Bars, 10 µm. (**C**) dsDNA release assay. Quantification of released dsDNA in supernatants of activated mouse eosinophils was assessed using PicoGreen fluorescent dye following the GM-CSF/C5a treatment in a time-dependent manner (*n* = 4). The dashed line indicates the mean level of spontaneously released dsDNA after 60 min eosinophil culture in the absence of stimulation. (**D**) Confocal microscopy. Mouse eosinophils were primed with GM-CSF and activated with C5a for 30 min. A representative image of EET formation, indicated by white arrows, is shown. Mitochondria, EPX and nuclei were stained with MitoSOX Red, monoclonal mouse anti-EPX antibody and Hoechst 33342, respectively. For better visualization of EETs, the gamma correction function together with min/max thresholds of Imaris software was used to optimize the image display by intensifying the grey value of red (mtDNA) and green (EPX) channels. Bars, 10 µm. Values are means ± SEM. *** *p* < 0.001; **** *p* < 0.0001.

As human and mouse eosinophils might exhibit different functional responses, we performed in vitro experiments on eosinophils purified from hypereosinophilic *Il5*^tg^ mice. To investigate the degranulation pattern, we stimulated mouse eosinophils in the same way as human eosinophils. Owing to constantly high levels of IL-5 in the blood of *Il5*^tg^ mice, the IL-5Rα expression is downregulated [39]. Therefore, we used GM-CSF as a priming agent for mouse eosinophils. Similar to the degranulation results obtained in the human system, mouse eosinophils completely degranulated within 1 min after C5a stimulation (Figure 4A). Notably, the extent of released EPX was lower in mouse (70–80%) (Figure 4A) compared to human eosinophils (80–90%) (Figure 3A). We then assessed the intracellular EPX content in mouse eosinophils by confocal microscopy and stained mitochondria, EPX, cell membrane and the nucleus of isolated mouse eosinophils using Mitotracker, anti-EPX antibody, anti-Siglec-F antibody and Hoechst 33342, respectively. Again, the amount of intracellular EPX was significantly decreased already 1 min after C5a stimulation (Figure 4B). In agreement with human data (Figure 3B), no further decrease in the intracellular EPX content was observed until 30 min of C5a stimulation (Figure 4B).

We also explored the kinetics of mtDNA release in the supernatant of C5a-stimulated mouse eosinophils and compared it to the degranulation pattern. A significant increase in mtDNA release by mouse eosinophils was observed 10 min after C5a stimulation (Figure 4C). Similar to human eosinophils (Figure 3C), we observed a continuous extracellular mtDNA release, which did not reach maximal levels before 60 min (Figure 4C).

To investigate the association of granule protein EPX with the released mtDNA, isolated mouse eosinophils were stained with anti-EPX antibody and MitoSOX following GM-CSF priming and subsequent C5a stimulation. As in the human system, we observed co-localization between EPX (green) and released mtDNA (red) in the extracellular space (white arrows) (Figure 4D), suggesting the formation of EETs. Taken together, mouse and human eosinophils exhibit similar characteristics in terms of degranulation, mtDNA release and EET formation.

## 4. Discussion

Eosinophils are a subset of granulocytes, which play an important role in the pathology of many allergic and non-allergic inflammatory diseases [40]. Upon appropriate activation, they can release granule proteins in a process known as degranulation. In the last few years, different stimuli have been described to induce different types of degranulation responses. However, the eosinophil degranulation kinetics has not been precisely investigated. We tested different physiological and non-physiological stimuli to select the best degranulation inducers for further experimentation. As monitored by surface CD63 expression and EPX release, eosinophil degranulation was most efficient in IL-5-primed and C5a-stimulated eosinophils. In agreement with previously published work, we observed relative inefficient eosinophil degranulation following PMA stimulation [37].

It is not clear whether C5a stimulation following IL-5 priming induces PMD or exocytosis in eosinophils. Based on ultrastructural studies, both eotaxin and PAF were reported to induce PMD [8]. The cell surface distribution and intracellular trafficking of CD63 within human eosinophils occur in both exocytosis and PMD [41]. Most previous studies have measured the release of granule proteins after longer time periods of stimulation such as 30 min [37,42], 1 h [41], 3 h [10] or even 6 h [43]. EPX and another granule-stored product, β-hexosaminidase, were detected in the supernatants of IFN-γ-stimulated eosinophils as early as 10 min but continued to increase up to 240 min [44]. Similarly, the amount of released MBP increased consistently during a 4 h incubation time with PMA [38]. Another kinetic study demonstrated a time-dependent increase in EDN release, reaching a plateau at 4 h of IL-5 stimulation [45]. Based on these earlier studies, we did not expect a complete EPX release within 1 min after C5a stimulation. On the other hand, one earlier study reported rapid activation of eosinophils by intracellular calcium mobilizing agonists [46]. In this study, we have not determined the type of degranulation, PMD or exocytosis that is induced in C5a-stimulated and cytokine-primed eosinophils. Therefore, it would be interesting to study important events of PMD, such as the formation of eosinophil Sombrero vesicles [7], and the underlining molecular mechanism in a time-dependent manner in subsequent studies.

Differences between human and mouse eosinophils have been described at different levels, including cell morphology, ultrastructure of their specific granules, types and amounts of cationic and other major proteins [47]. As compared to human eosinophils, we observed a decreased proportion of mouse eosinophils releasing EPX, a finding that might be related to the source of mouse eosinophils (hypereosinophilic *Il5*^tg^ mice). It is possible that the degranulation resistant eosinophils belong to another eosinophil subpopulation with a different transcriptome and that the size of this subpopulation differs between mice [48] and humans [49]. However, independent of this possible small inter-species difference, we demonstrate similar degranulation kinetics in human and mouse eosinophils following cytokine priming and subsequent C5a stimulation.

In the context of EETs, granule proteins are associated with an mtDNA scaffold in the extracellular space [13]. When the association between granule protein and mtDNA occurs remains unknown. It has been previously demonstrated that single eosinophils release mtDNA in less than 1 sec, but the time point of release differs between different eosinophils [13]. Interestingly, the kinetics of EPX and mtDNA release differs when assessed in a bulk eosinophil population. While EPX release reaches its maximum already after 1 min, extracellular mtDNA levels continue to increase for up to 30–60 min. These data imply that many single eosinophils release EPX first before they release mtDNA, suggesting different molecular mechanisms responsible for their secretions.

The question of why eosinophils form an mtDNA scaffold with a significant time delay after degranulation remains to be resolved. We speculate that the rapid release of the preformed granule proteins [50,51], as shown in this study, represents an unfocused response to fight against invading pathogens that is associated with damage of the surrounding tissues [52]. To be more efficient, granule proteins are then located within EETs, which have been shown to bind to and kill bacteria [13]. The recruitment of granule proteins to the DNA scaffold secures a high concentration of granule proteins in close proximity to the pathogens, and it is likely that in such a scenario tissue, damage is limited.

Taken together, we provide evidence that the process of degranulation is rapid and occurs independently of mtDNA release. The mtDNA released from eosinophils might encounter and bind already to released EPX in the extracellular space. Alternatively, negatively charged mtDNA might be released through previously fused, emptied granules and interact with remaining intragranular proteins to form EETs. The exact mechanism of EET formation remains unknown and requires further investigation. A better understanding of EET formation might enhance the comprehension of eosinophil-related allergic and non-allergic inflammatory diseases.

## Data Availability

All data presented in this study are available on request from the corresponding author.

## Abbreviations

APC, allophycocyanin; AU, arbitrary unit; BSA, bovine serum albumin; C5a, complement factor 5a; dsDNA, double-stranded DNA; DTT, dithiothreitol; ECP, eosinophil cationic protein; EDN, eosinophil-derived neurotoxin; EET, eosinophil extracellular trap; EoSV, eosinophil Sombrero vesicle; EPX, eosinophil peroxidase; FCS, fetal calf serum; GAPDH, glyceraldehyde 3-phosphate dehydrogenase; GM-CSF, granulocyte/macrophage colony-stimulating factor; HRP, horseradish peroxidase; IFN-γ, interferon-γ; IL-5, interleukin-5; MBP, major basic protein; MFI, mean fluorescence intensity; mtDNA, mitochondrial DNA; NET, neutrophil extracellular trap; OPD, o-phenylenediamine; PAF, platelet-activating factor; PBMC, peripheral blood mononuclear cell; PBS, phosphate buffered saline; PE, phycoerythrin; PMA, phorbol 12-myristate 13-acetate; PMD, piecemeal degranulation; PMSF, phenylmethylsulphonyl fluoride; RFU, relative fluorescence unit; RT, room temperature; tg, transgenic.
